# Supplementary material for: Genome-Wide Analysis of the Auxin/Indoleacetic Acid (Aux/IAA) Gene Family in Autopolyploid Sugarcane (Saccharum spontaneum)
Source: Int J Mol Sci. 2024 Jul 8;25(13):7473. doi: 10.3390/ijms25137473 (PMC11242263; doi:10.3390/ijms25137473)
Supplement: Supplementary file 1 [file ijms-25-07473-s001.zip › ijms-3069290-supplementary.pdf]

International Journal of Molecular Sciences

MDPI

Supplementary Data

Table S1. SsIAA gene family in sugarcane *Saccharum spontaneum*.

Gene name	Gene ID	Chromosome	n(IAA)	MV (kD)	PI	AI	Subcellular localization	Gene duplication	Position on the genome	Strand Orientation	Annotations
SsIAA1	Sspon.02G0041760-1B	Chr2B	783	82.65	9.44	64.91	Nucleus.	Dispersed	77885470-77900915	negative	No GO associations found
SsIAA2a	Sspon.03G0022760-1A	Chr3A	204	21.05	5.38	48.17	Nucleus.	Dispersed	69521520-69522442	positive	AUX/IAA domain
SsIAA2b	Sspon.03G0022760-2B	Chr3B	239	24.66	6.97	50.91	Nucleus.	Unknown	91938691-91939948	negative	Regulation of transcription, DNA-templated
SsIAA2c	Sspon.03G0022760-3C	Chr2C	239	24.66	6.97	50.91	Nucleus.	WGD or Segmental	81934635-81935892	negative	Regulation of transcription, DNA-templated
SsIAA3a	Sspon.03G0024820-1A	Chr3A	342	36.48	9.34	44.68	Nucleus.	Dispersed	75376437-75380644	positive	Response to auxin
SsIAA3b	Sspon.03G0024820-2B	Chr3B	252	27.05	8.96	44.3	Nucleus.	WGD or Segmental	98880079-98883120	negative	Fruit Development
SsIAA3c	Sspon.03G0024820-3C	Chr3C	265	28.64	6.67	37.48	Nucleus.	WGD or Segmental	93967884-93971621	negative	Response to auxin
SsIAA4a	Sspon.05G0001870-1A	Chr5A	947	104.19	5.87	61.16	Nucleus.	Dispersed	5939018-5943933	positive	Root development
SsIAA4d	Sspon.05G0001870-3D	Chr5D	752	82.53	5.33	63.88	Nucleus.	WGD or Segmental	3857874-3862563	positive	Regulation of transcription, DNA-templated
SsIAA5.1a	Sspon.03G0011430-1A	Chr3A	589	62.87	10.52	52.91	Nucleus.	Dispersed	31092210-31096179	positive	Regulation of transcription, DNA-templated
SsIAA5.1d	Sspon.03G0041260-2D	Chr3D	275	28.87	6.74	58.37	Nucleus.	Dispersed	29857201-29857559	negative	Regulation of transcription, DNA-templated
SsIAA6	Sspon.03G0006790-1A	Chr3A	388	42.06	9.15	44.89	Nucleus.	WGD or Segmental	18637083-18642587	negative	Response to heat
SsIAA7a	Sspon.04G0014810-1A	Chr4A	278	29.59	7.8	49.33	Nucleus.	Tandem	55340442-55343662	positive	Response to auxin
SsIAA7p	Sspon.04G0014810-1P	Chr4A	241	25.5	5.21	49.18	Nucleus.	Singleton	55326818-55327075	positive	Regulation of transcription, DNA-templated
SsIAA7b	Sspon.04G0014810-2B	Chr4B	249	26.31	4.98	46.89	Nucleus.	WGD or Segmental	58183278-58186621	negative	Regulation of transcription, DNA-templated
SsIAA7d	Sspon.04G0014810-3D	Chr4D	276	29.12	5.57	48.38	Nucleus.	WGD or Segmental	77885470-77900915	negative	No GO associations found
SsIAA8b	Sspon.08G0022500-1B	Chr8B	809	88.67	5.42	66.21	Nucleus.	Unknown	49305894-49314316	negative	Response to hormone
SsIAA8d	Sspon.08G0022500-3D	Chr8D	793	85.86	5.71	54.34	Chloro-plast.	Unknown	51741750-51759860	negative	Protein Folding

SsIAA9a	Sspon.04G0001230-1A	Chr4A	191	20.44	5.49	42.22	Nucleus.	WGD or Segmental	4697296-4698075	negative	Regulation of transcription, DNA-templated
SsIAA9c	Sspon.04G0001230-2C	Chr4C	191	20.47	5.67	44.23	Nucleus.	WGD or Segmental	4736674-4737453	positive	Regulation of transcription, DNA-templated
SsIAA9d	Sspon.04G0001230-3D	Chr2D	193	20.66	5.78	41.44	Nucleus.	Dispersed	6668660-6669446	positive	Regulation of transcription, DNA-templated
SsIAA10	Sspon.06G0002150-1T	Chr6A	1429	155.9	5.77	57.27	Nucleus.	Singleton	6930821-6932644	negative	Response to hormone
SsIAA11a	Sspon.01G0023790-1A	Chr1A	330	34.98	5.29	64.09	Nucleus.	WGD or Segmental	85454695-85457182	positive	Regulation of transcription, DNA-templated
SsIAA11b	Sspon.01G0023790-2B	Chr1B	298	31.23	6.03	66.53	Nucleus.	WGD or Segmental	90999503-91002075	negative	Regulation of transcription, DNA-templated
SsIAA11c	Sspon.01G0023790-3C	Chr1C	242	25.36	4.83	58.63	Nucleus.	WGD or Segmental	84253686-84256000	negative	Regulation of transcription, DNA-templated
SsIAA12.1a	Sspon.01G0023800-1A	Chr1A	217	23.1	8.7	48.53	Nucleus.	Singleton	85497372-85498224	positive	Regulation of transcription, DNA-templated
SsIAA12.2p	Sspon.01G0023800-1P	Chr2A	232	24.59	8.6	56.09	Nucleus.	Dispersed	111442811-111443713	positive	Regulation of transcription, DNA-templated
SsIAA12.1b	Sspon.01G0023800-2B	Chr1B	217	23.1	8.7	48.53	Nucleus.	Proximal	90932535-90933387	negative	Regulation of transcription, DNA-templated
SsIAA13a	Sspon.01G0028160-1A	Chr1A	234	25.18	8.86	48.73	Nucleus.	WGD or Segmental	90932535-90933387	negative	Regulation of transcription, DNA-templated
SsIAA13c	Sspon.01G0028160-3C	Chr1C	234	25.22	8.86	48.73	Nucleus.	Dispersed	50703191-50705230	positive	Negative regulation of transcription, DNA-templated
SsIAA14	Sspon.02G0041760-3D	Chr1D	175	18.8	6.73	35.71	Nucleus.	Dispersed	101374456-101375168	positive	Regulation of transcription, DNA-templated
SsIAA15b	Sspon.03G0027750-1B	Chr3B	748	83.41	7.03	56.43	Nucleus.	WGD or Segmental	6658010-6662331	negative	Regulation of transcription, DNA-templated
SsIAA15c	Sspon.03G0027750-2C	Chr3C	783	87.67	5.61	58.4	Nucleus.	WGD or Segmental	10437460-10441553	negative	Regulation of transcription, DNA-templated
SsIAA15d	Sspon.03G0027750-3D	Chr3D	844	94.51	6.87	57.85	Nucleus.	Dispersed	5479687-5483759	positive	Regulation of transcription, DNA-templated
SsIAA15p	Sspon.03G0027750-1P	Chr3C	798	89.36	5.75	58.77	Nucleus.	WGD or Segmental	10105959-10110054	positive	Regulation of transcription, DNA-templated
SsIAA16	Sspon.03G0022760-1P	Chr7B	233	23.99	6.76	42.19	Nucleus.	Dispersed	45460339-45461913	negative	Regulation of transcription, DNA-templated
SsIAA17a	Sspon.07G0010810-1A	Chr7A	248	26.71	7.68	41.01	Nucleus.	Unknown	35935896-35938803	positive Family	Regulation of transcription, DNA-templated
SsIAA17b	Sspon.07G0010810-2B	Chr7B	248	26.6	7.65	40.96	Nucleus.	Unknown	38800636-38803570	negative	Regulation of transcription, DNA-templated
SsIAA17d	Sspon.07G0010810-3D	Chr7D	248	26.7	7.68	40.31	Nucleus.	Unknown	32690862-32693773	negative	Regulation of transcription, DNA-templated

SsIAA18a	Sspon.07G0003900-1A	Chr7A	359	38.42	5.84	52.94	Nucleus.	Unknown	9728691-9733926	positive	Regulation of transcription, DNA-templated
SsIAA18b	Sspon.07G0003900-2B	Chr7B	316	33.9	5.2	54.61	Nucleus.	Unknown	8805641-8807419	positive	Regulation of transcription, DNA-templated
SsIAA18c	Sspon.07G0003900-3C	Chr7C	354	38.03	5.3	52.1	Nucleus.	Unknown	5991219-5993483	negative	Regulation of transcription, DNA-templated
SsIAA18d	Sspon.07G0003900-4D	Chr7D	346	37.2	5.49	52.76	Nucleus.	Unknown	8910757-8913013	positive	Regulation of transcription, DNA-templated
SsIAA18t	Sspon.07G0003900-1T	Chr7B	343	36.92	5.3	54.89	Nucleus	Unknown	8809315-8811569	positive	Regulation of transcription, DNA-templated
SsIAA19a	Sspon.07G0002380-1A	Chr7A	267	27.95	6.2	47.95	Nucleus.	Unknown	5913783-5917916	positive	Regulation of transcription, DNA-templated
SsIAA19b	Sspon.07G0002380-2B	Chr7B	268	28.01	6.2	50.17	Nucleus.	Unknown	4333997-4338131	positive	Regulation of transcription, DNA-templated
SsIAA19d	Sspon.07G0002380-3D	Chr7D	317	33.4	6.83	49.42	Nucleus.	Unknown	5092603-5096942	negative	Regulation of transcription, DNA-templated
SsIAA19t	Sspon.07G0002380-1T	Chr7D	319	33.54	6.83	51.58	Nucleus.	Unknown	5104197-5108531	negative	Regulation of transcription, DNA-templated
SsIAA20	Sspon.08G0013920-1A	Chr8A	189	20.54	5.99	60	Nucleus.	Unknown	57584849-57586099	positive	Regulation of transcription, DNA-templated
SsIAA21.1a	Sspon.08G0007190-1A	Chr8A	379	42.07	8.99	38.47	Nucleus.	Unknown	22454558-22466916	positive	Response to heat
SsIAA21.2b	Sspon.08G0020950-1B	Chr8B	499	54.47	8.83	42.37	Nucleus.	Unknown	28475384-28488002	positive	Response to heat
SsIAA21.2c	Sspon.08G0020950-2C	Chr8C	247	26.97	9.02	41.71	Nucleus.	Unknown	30712978-30715935	negative	Response to heat
SsIAA22	Sspon.03G0037540-1B	Chr3B	639	71.77	5.72	58.42	Nucleus.	WGD or Segmental	99443945-99447482	positive	Regulation of transcription, DNA-templated
SsIAA23	Sspon.03G0036500-1B	Chr3B	214	23.15	7.77	48.78	Nucleus.	WGD or Segmental	90332265-90334872	negative	Regulation of transcription, DNA-templated
SsIAA24a	Sspon.02G0026740-1A	Chr2A	191	20.4	6.31	38.52	Nucleus.	Dispersed	94923088-94923850	positive	Regulation of transcription, DNA-templated
SsIAA24d	Sspon.02G0026740-2D	Chr2D	193	20.69	6.3	36.91	Nucleus.	WGD or Segmental	72560428-72561194	negative	Regulation of transcription, DNA-templated
SsIAA24c	Sspon.02G0041760-2C	Chr2C	191	20.54	5.94	38	Nucleus.	WGD or Segmental	82233599-82234359	negative	Regulation of transcription, DNA-templated
SsIAA25b	Sspon.02G0027120-2B	Chr2B	768	85.17	5.97	64.77	Nucleus.	WGD or Segmental	95779585-95785095	negative	Regulation of transcription, DNA-templated
SsIAA25c	Sspon.02G0027120-3C	Chr2C	831	91.82	6.1	57.56	Nucleus.	Tandem	123917001-123922379	positive	Regulation of transcription, DNA-templated
SsIAA25d	Sspon.02G0027120-4D	Chr2D	823	91.3	6.37	57.92	Nucleus.	WGD or Segmental	108412153-108417783	positive	Regulation of transcription, DNA-templated

SsIAA26a	Sspon.02G0009510-1A	Chr2A	139	15.24	4.62	32.78	Nucleus.	WGD or Segmental	26751431-26752199	negative	Regulation of transcription, DNA-templated
SsIAA25t	Sspon.02G0027120-1T	Chr2C	828	91.42	6.08	54.82	Nucleus.	WGD or Segmental	123858266-123863523	negative	Regulation of transcription, DNA-templated
SsIAA26d	Sspon.02G0009510-2D	Chr2D	762	82.5	10.87	60.42	Nucleus.	WGD or Segmental	21321854-21325253	negative	Regulation of transcription, DNA-templated
SsIAA27a	Sspon.05G0016400-1A	Chr5A	162	17.22	5.87	34.82	Nucleus.	Singleton	66902764-66903857	positive	Regulation of transcription, DNA-templated
SsIAA27d	Sspon.05G0016400-3D	Chr5D	363	38.28	6.36	47.54	Nucleus.	WGD or Segmental	61679853-61685979	negative	Regulation of transcription, DNA-templated
SsIAA28a	Sspon.02G0031340-1A	Chr2A	878	96.97	5.94	64.59	Nucleus.	WGD or Segmental	114616279-114621376	positive	Regulation of transcription, DNA-templated
SsIAA28b	Sspon.02G0031340-2B	Chr2B	954	105.41	5.99	66.1	Nucleus.	WGD or Segmental	108396410-108402701	negative	Regulation of transcription, DNA-templated
SsIAA28d	Sspon.02G0059280-1D	Chr2D	682	75.35	5.46	57.99	Nucleus.	WGD or Segmental	91038425-91043610	negative	Regulation of transcription, DNA-templated
SsIAA29	Sspon.04G0010200-2B	Chr4B	668	74.42	5.89	61.13	Nucleus.	WGD or Segmental	26207214-26212018	negative	Negative regulation of transcription, DNA-templated
SsIAA30-1p	Sspon.01G0023790-1P	Chr2A	288	30.28	5.18	57.26	Nucleus.	WGD or Segmental	111385647-111390381	positive	Regulation of transcription, DNA-templated
SsIAA30-2p	Sspon.01G0023790-2P	Chr2C	288	30.23	5.18	57.26	Nucleus.	Dispersed	108500009-108505471	positive	Regulation of transcription, DNA-templated
SsIAA31c	Sspon.01G0023800-3C	Chr2C	226	24.03	8.24	54.97	Nucleus.	Dispersed	108456291-108457177	negative	Regulation of transcription, DNA-templated
SsIAA31t	Sspon.01G0023800-1T	Chr2C	226	24.03	8.34	54.97	Nucleus.	Dispersed	108443679-108444565	negative	Regulation of transcription, DNA-templated
SsIAA32b	Sspon.05G0028050-1B	Chr5B	655	72.49	5.87	59.41	Nucleus.	Dispersed	60122402-60128086	positive	Regulation of transcription, DNA-templated
SsIAA32d	Sspon.05G0028050-2D	Chr5D	497	55.15	8.21	54.27	Nucleus.	WGD or Segmental	33290107-33295837	negative	Regulation of transcription, DNA-templated
SsIAA32p	Sspon.05G0028050-1P	Chr5B	614	68.56	6.56	62.07	Nucleus.	Singleton	25637110-25642730	negative	Regulation of transcription, DNA-templated
SsIAA33.1c	Sspon.06G0032700-1C	Chr6C	828	90.87	6.6	52.21	Nucleus.	Proximal	91761546-91766954	positive	Regulation of transcription, DNA-templated
SsIAA33.2d	Sspon.06G0032710-2D	Chr6D	623	69.17	8.3	50.02	Nucleus.	Unknown	60837204-60840346	positive	Regulation of transcription, DNA-templated
SsIAA34.1-1p	Sspon.04G0017640-1P	Chr8A	901	99.99	5.93	68.72	Nucleus.	Unknown	4675614-4680912	negative	Response to hormone
SsIAA34.1-2p	Sspon.04G0017640-2P	Chr8A	907	100.28	5.98	69.97	Nucleus.	Unknown	10431374-10436702	positive	Regulation of transcription, DNA-templated
SsIAA34.2b	Sspon.04G0017640-2B	Chr4B	937	103.78	5.91	66.57	Nucleus.	Unknown	67836574-67841376	positive	Regulation of transcription, DNA-templated

SsIAA34.2-3p	Sspon.04G0017640-3P	Chr8B	918	101.62	5.86	68.94	Nucleus.	WGD or Segmental	7423247-7428567	positive	Regulation of transcription, DNA-templated
SsIAA34.3d	Sspon.08G0025580-2D	Chr8D	783	85.69	5.66	61.19	Nucleus.	Unknown	9533170-9538484	positive	Regulation of transcription, DNA-templated
SsIAA35.1p	Sspon.04G0018530-1P	Chr8A	899	99.48	6.36	57.25	Nucleus.	Unknown	6040168-6047060	negative	Regulation of transcription, DNA-templated
SsIAA35.2c	Sspon.08G0025000-1C	Chr8C	988	108.65	6.14	57.85	Nucleus.	Unknown	4581592-4589038	positive	Regulation of transcription, DNA-templated
SsIAA36a	Sspon.04G0018530-1A	Chr4A	1867	207.23	6.25	57.06	Nucleus.	WGD or Segmental	66589636-66611591	negative	Regulation of transcription, DNA-templated
SsIAA36c	Sspon.04G0018530-3C	Chr4C	798	88.97	6.13	51.02	Nucleus.	WGD or Segmental	72152308-72156978	positive	Regulation of transcription, DNA-templated
SsIAA36d	Sspon.04G0018530-4D	Chr4D	1904	211.59	5.98	57.89	Nucleus.	WGD or Segmental	75890162-75912875	positive	Regulation of transcription, DNA-templated
SsIAA37.1b	Sspon.04G0030300-1B	Chr4B	883	98.89	5.48	51.09	Nucleus.	Dispersed	76690693-76697801	negative	Regulation of transcription, DNA-templated
SsIAA37.2c	Sspon.04G0034270-1C	Chr4C	762	84.68	5.9	56.71	Nucleus.	Tandem	72159880-72166361	positive	Response to hormone
SsIAA38c	Sspon.06G0019860-2C	Chr6C	1091	120.82	6.14	57.95	Nucleus.	Dispersed	5171377-5177422	positive	Response to hormone
SsIAA38d	Sspon.06G0019860-3D	Chr6D	1095	121.59	6.41	58.4	Nucleus.	WGD or Segmental	3990976-3996960	positive	Response to hormone

Abbreviations: MV=molecular weight, nIAA=number of amino acids, pI=isoelectric point, AI=aliplatic index.

Figure S1. Ten conserved motif sequences and logos were identified in SsIAA proteins.

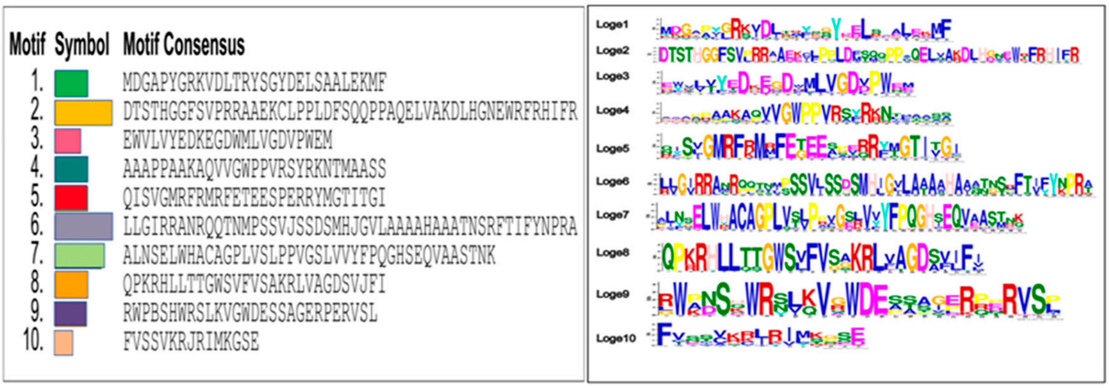

Table S2. Summary of cis-acting elements types, functional classification, and counts in 92 *SsIAAs* promoters.

Serial number	Name of element	Components correspond to functional classification	Quantity	Total of similar functions
		**Hormonal elements correspond to functions**		
1	TGACG-motif	MeJA-responsiveness	214	428
2	CGTCA-motif	MeJA-responsiveness	214	
3	TGA-element	auxin responsiveness	69	104
4	GARE-motif	auxin responsiveness	28	
5	AuxRR	auxin responsiveness	7	
6	P-box	gibberellin-responsive	49	67
7	TATC-box	gibberellin-responsiveness	18	
8	TCA-element	salicylic acid responsiveness	33	33
		**Stress Responsiveness**		
9	ABRE	anaerobic induction	387	539
10	ARE	anaerobic induction	152	
11	GC-motif	anoxic specific inducibility	91	91
12	LTR	low-temperature responsiveness	83	83
13	MBS/MBSI	drought-inducibility	64	64
14	TC-rich repeats	stress responsiveness	15	15
		**Growth and development class elements**		
15	CAT-box	meristem expression	97	97
16	RY-element	seed-specific regulation	24	24
17	MRE	light responsiveness	18	50
18	ACE	light responsiveness	16	
19	ATCT-motif	light response	16	
32	ATC-motif	light responsiveness	1	
20	GCN4_motif	endosperm expression	14	14
21	circadian	circadian control	10	10
22	HD-Zip	differentiation of the palisade mesophyll cells	6	6

23	AACA_motif	endosperm-specific negative expression	1	1
		**Transcriptional activation elements**		
24	CAAT-box	promoter and enhancer regions	2043	2043
25	TATA-box	transcription start	1931	1931
26	MYB	MYBHv1 binding site/Myb-binding site/MYB-like sequence/MYB recognition site/	675	753
28	CCAAT-box	MYB binding site involved in flavonoid biosynthetic gene regulation	78	
27	A-box	core promoter element around -30 of transcription start	163	163
29	TC-rich repeats	stress responsiveness	15	15
30	MSA-like	cell cycle regulation	15	15
31	AT-rich element	DNA binding protein (ATBP-1)	7	7
33	AACA_motif	endosperm-specific negative expression	1	1

Figure S2. Transcritome data of 38 *SsIAAs* in FPKMs.

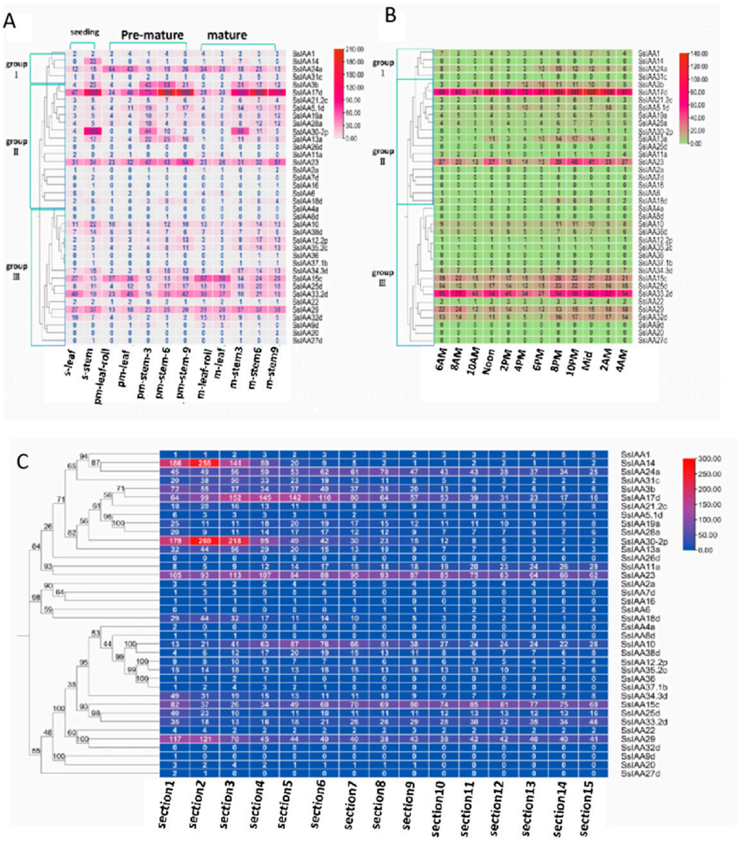

(A) Tissue-specific expressions. **(B)** Circadian rhythms. **(C)** Leaf sections.

Table S3. Primer sequences for qPCR.

Primer name	Sequence（5′-3′）	Gene ID
SsAUX/IAA2-F	CAAGAAGCGCGACAGCAAC	Sspon.01G0023800
SsAUX/IAA2-R	GTCCATGCTCACCTTCACGA	Sspon.01G0023800
SsAUX/IAA3-F	GAACGTCATGACCGTCCAGT	Sspon.01G0028160
SsAUX/IAA3-R	AGAGCGATGGAGAGGTCCTT	Sspon.01G0028160
SsAUX/IAA6-F	ACAATCAGCTGTTCTCGGCA	Sspon.02G0027120
SsAUX/IAA6-R	GTGATTGGCTTGTTGGGCTG	Sspon.02G0027120
SsAUX/IAA15-F	TGAGGTCGTACCGCAAGAAC	Sspon.03G0036500
SsAUX/IAA15-R	GGCGAACTTCCTGATGGTGA	Sspon.03G0036500
SsAUX/IAA18-F	GAAGAGAGGGTTCGCGGAAG	Sspon.04G0001230
SsAUX/IAA18-R	ATGGCGTCCCCTTCCTTCTT	Sspon.04G0001230
SsAUX/IAA19-F	TCTCCGTGGGCTAACTCTGA	Sspon.04G0010200
SsAUX/IAA19-R	AAAGGAGGTTGAGGTGGCTG	Sspon.04G0010200
SsAUX/IAA20-F	TACAGAGAATGCCCGGCTTG	Sspon.04G0014810
SsAUX/IAA20-R	GGCTGCCTTCTTCCCATTCT	Sspon.04G0014810
SsAUX/IAA30-F	CACAAGTTGTTGGATGGCCG	Sspon.07G0002380
SsAUX/IAA30-R	GCTCCATCCATGCTGACCTT	Sspon.07G0002380
SsAUX/IAA31-F	AGAGGAAGAAAGGGTGCTGC	Sspon.07G0003900
SsAUX/IAA31-R	TCTTGCAAGTTTGCTTCGCC	Sspon.07G0003900
Q-Ss.GADPH-F	CACGGCCACTGGAAGCA	control
Q-Ss.GADPH-R	TCCTCAGGGTTCCTGATGCC	control

Table S4. Primer sequences for cloning of SsIAA-GFP fusion constructs.

Primer name	Sequences
35s-SsIAA2-2F	acgggggacgagctcggtaccATGGAGGTCGCCGCAGCA
35s-SsIAA2-2R	tctagaggatccccgggtaccGTTGTTCTTGACTGAGCCGAGC
35s-SsIAA15-F	acgggggacgagctcggtaccATGTCGGTGGAGACGGAGC
35s-SsIAA15-R	tctagaggatccccgggtaccTCGGGCAGCTCTTGGTGC
